# How to recover from a bad start: adaptation of HIV-1 transcription start site mutants during serial passaging in culture

**DOI:** 10.1128/jvi.00159-25

**Published:** 2025-05-07

**Authors:** Olga A. Nikolaitchik, Akhil Chameettachal, Saiful Islam, Zetao Cheng, Krista Delviks-Frankenberry, Brandon F. Keele, Vinay K. Pathak, Wei-Shau Hu

**Affiliations:** 1 Viral Recombination Section, Frederick, Maryland, USA; 2 Viral Mutation Section, HIV Dynamics and Replication Program, National Cancer Institute at Frederick 585796 , Frederick, Maryland, USA; 3 AIDS and Cancer Virus Program, Frederick National Laboratory for Cancer Research, Frederick, Maryland, USA; Icahn School of Medicine at Mount Sinai, New York, New York, USA

**Keywords:** HIV, transcription, RNA, genome packaging, translation, replication fitness, reverse transcription, transcription start sites

## Abstract

HIV-1 uses neighboring sequences as transcription start sites and generates multiple unspliced transcripts, including two major transcripts with three guanosines (3G) or one guanosine (1G) at the 5′ end. Although only differing by 2-nt, 3G RNA and 1G RNA are functionally distinct. We have previously generated two mutants: the TTG virus mainly expresses 1G RNA, whereas the plusAC virus predominantly expresses 3G RNA. Both mutant viruses are replication-competent but exhibit fitness defects. Here, we passaged the plusAC virus in T cells and characterized the changes near the transcription start sites. We observed the rapid loss of the plusAC virus genotype and the emergence of multiple revertants. All major revertants that dominated the cultures had a 1- to 3-nt deletion that compensated for the dinucleotide insertion in the plusAC virus. These major revertants express more than one major transcript, preferentially package 1G RNA, and have improved replication kinetics compared with the plusAC virus. Most major revertants likely arose through errors during reverse transcription, including misalignments during minus-strand DNA transfer, nucleotide deletion in a homopolymer run, or deletion of a short direct repeat. Additionally, we have determined that a T-to-G substitution near transcription start sites occurs at ~5% per replication cycle by copying the guanosine cap. Converting a base to guanosine through cap copying has been observed in multiple positions, but always directly upstream of a major transcription start site. Taken together, our findings demonstrate the selection pressure for expressing functionally distinct unspliced RNA species to optimize replication fitness.

**IMPORTANCE:**

HIV-1 unspliced RNA serves as the mRNA to translate Gag/Gag-Pol polyproteins and as the virion genome. HIV-1 produces two major RNA species: 1G RNA is preferentially packaged and 3G RNA is favorably translated, although each transcript can perform both functions. We have previously generated a replication-competent mutant virus that mainly expresses 3G RNA and observed that this mutant has replication fitness defects. We found that the mutant virus improved its replication kinetics after passaging, indicating adaptation. Our analyses showed that, through mutations occurring during DNA synthesis, multiple revertants arose rapidly to replace the input mutant virus. The major revertants regained the ability to generate more than one major transcript and preferentially package 1G RNA. These results highlight the importance of expressing HIV-1 RNA species that serve distinct functions and the ability of HIV-1 to adapt through mutations in the genome.

## INTRODUCTION

A member of the Retroviridae, HIV-1 packages unspliced RNA into virions, and upon infection of target cells, reverse transcribes the RNA into DNA. HIV-1 DNA is integrated into the host chromosome and becomes a provirus, which remains a permanent part of the host genome (1). The genetic structures of HIV-1 DNA and RNA differ at their ends. HIV-1 DNA has two long terminal repeats (LTRs), one at each end of the DNA; the LTR is divided into U3, R, and U5 regions. In contrast, HIV-1 RNA has R-U5 at the 5′ end and U3-R at the 3′ end, with the R region being the repeated sequence at the two ends. Thus, during DNA synthesis, the HIV-1 encoded enzyme reverse transcriptase (RT) must reconstruct the LTR and place a copy at each end of the DNA (2). This is accomplished by two obligatory strand transfer steps: minus- and plus-strand DNA transfer. DNA synthesis initiates near the 5′ end of the viral RNA and generates a short DNA known as the minus-strand strong-stop DNA, which contains a segment that is complementary to the R region at the 3′ end of the viral RNA. Facilitated by this complementarity, RT switches to use the 3′ end of RNA as the template in a step termed minus-strand DNA transfer and continues DNA synthesis. The minus-strand DNA transfer step reconnects U3-R-U5 and reconstructs the LTR. The LTR is duplicated in the plus-strand DNA transfer step, and upon completion of DNA synthesis, a DNA copy with two LTRs is generated (2).

Host RNA polymerase II (Pol II) recognizes the HIV-1 promoter located in U3 and transcribes the provirus to generate HIV-1 RNA. Similar to cellular mRNA, HIV-1 transcripts are modified by the host machinery to include a modified 5′ guanosine cap as well as a polyA tail (1). Some HIV-1 transcripts undergo complex alternative splicing to express multiple viral genes, whereas other HIV-1 transcripts remain unspliced (3). The unspliced, full-length HIV-1 RNA (referred to as HIV-1 RNA hereafter) has two distinct roles: it serves as an mRNA for the translation of Gag and Gag-Pol polyproteins, and it also serves as the virion genome to transmit genetic information to the progeny (4–6). How HIV-1 RNA serves these two distinct roles has been a long-standing question in the field.

Recent studies have revealed that Pol II uses neighboring sequences as transcription start sites and generates multiple species of HIV-1 RNA that vary by a few nucleotides (nts) at the 5′ end (7–9). There are three consecutive guanosines at the junction of U3 and R. For clarity, these three guanosines are also referred to as the start of the R region in the text below. Transcription can initiate at any of these guanosines, producing HIV-1 RNAs containing three, two, or one guanosine at the 5′ end, referred to as 3G, 2G, and 1G RNA, respectively. The 3G and 1G RNAs are the two major transcripts in the cells (7–10); they are identical 9 kb RNAs, except that 3G RNA has two additional guanosines at the 5′ end. However, these two RNAs differ functionally. Although both RNAs are present in the cells, 1G RNA is selected over 3G RNA for packaging into particles as virion genomes (7–9). RNA structural analyses have shown that the 5′ untranslated region (5′ UTR) of 3G and 1G RNA each folds into an ensemble of structures. The 1G RNA, but not the 3G RNA, tends to fold into structures that facilitate genome packaging, including exposed dimerization initiation signals and primary Gag binding sites, allowing RNA:RNA and RNA:Gag interactions (9). Both 3G and 1G RNA can be translated; however, 3G RNA is translated more efficiently than 1G RNA (11). Thus, the two major HIV-1 RNA species each excels at one of the two functions.

Our previous observations showed that HIV-1 transcription initiation is affected by the distance between the CATA/TATA box and the three consecutive guanosines, as well as the nucleotide composition of the major transcription start sites (12). Using this knowledge, we generated two HIV-1 mutants, each containing a 2-nt modification near the U3-R junction, which resulted in altered transcription start site usage. The TTG mutant contains two substitutions that change the three consecutive guanosines to TTG, and this mutant expresses mostly 1G RNA (12). The plusAC mutant has an AC dinucleotide insertion immediately upstream of the three consecutive guanosines, and this mutant expresses mostly 3G RNA (12). Our studies showed that although both plusAC and TTG viruses can undergo multiple rounds of replication in T cells, they both exhibit defects in replication fitness (12). These findings indicate that HIV-1 optimizes replication fitness by expressing multiple RNA species with specialized functions.

We hypothesized that an HIV-1 mutant that produces only one major transcript would adapt to overcome the defect caused by the imbalance of RNA functions from expressing single RNA species. We envision two different paths for adaptation. The mutant virus could revert to the wildtype phenotype and produce a mixture of 3G and 1G RNA. Alternatively, the mutant virus could adapt to use another RNA species to functionally replace either 1G or 3G RNA. In this report, we examined the adaptation of the plusAC virus during spreading infection in T cells. We observed that the plusAC virus rapidly adapted by changing into multiple genotypes. These findings provide insights into the regulation of HIV-1 transcription start sites and RNA functions.

## RESULTS

### Passaging of NL4-3-plusAC in T cells

**Fig 1:**
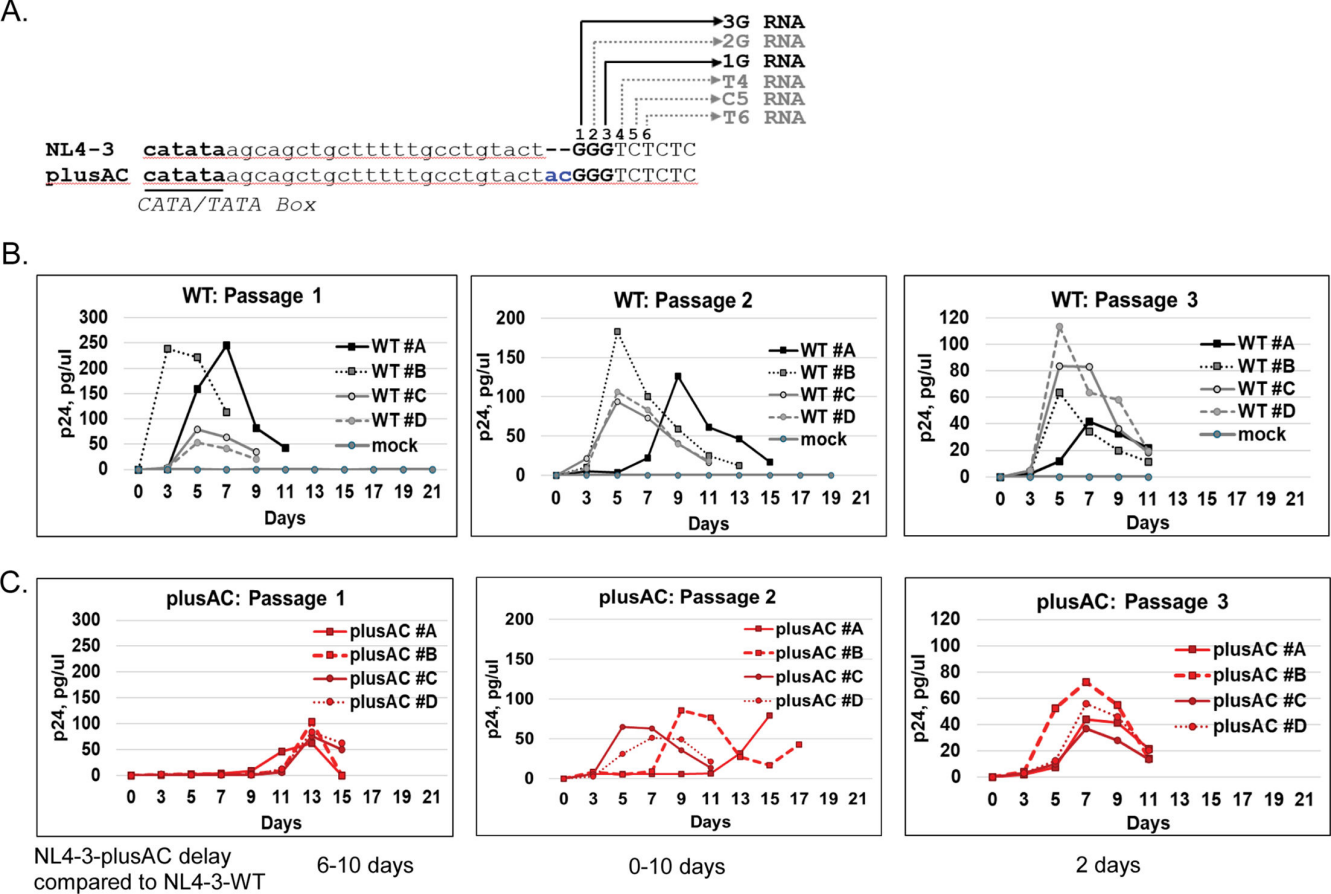
Serial passaging of wildtype (WT) and mutant NL4-3. (**A**) Sequences near U3-R junctions from NL4-3 and NL4-3-plusAC. U3 sequences are shown in lower case, whereas R sequences are shown in upper case. The overlapping CATA/TATA box is in bold and underlined, whereas the three consecutive guanosines are shown in bold. The nucleotide numbering on top of the sequence starts from the most 5′ nt of the three guanosines. The 2-nt insertion in NL4-3-plusAC is shown in blue. Transcription start sites for major RNA species (1G and 3G) are indicated by black arrows while grey arrows denote transcription starts for minor RNA species. Replication kinetics of NL4-3 (**B**) and NL4-3-plusAC (**C**) during serial passaging in Hut78/R5 cells. Viruses from the peak virus production time point were used to inoculate the next passage. Replication kinetics were monitored by p24 ELISA.

We have previously shown that with a 2-nt insertion, NL4-3-plusAC mostly expresses 3G RNA (Fig. 1A) and exhibits a defect in replication kinetics (12). To better understand the regulation of HIV-1 transcription initiation and RNA function, we examined the adaptation of NL4-3-plusAC virus during passaging experiments. We generated four viral stocks from different transfections with NL4-3 and NL4-3-plusAC and used each stock to initiate an independent culture experiment. Since NL4-3-plusAC has an RNA packaging defect (12), we infected Hut78/R5 cells with 25 ng p24 of NL4-3 (WT) or 50 ng p24 of NL4-3-plusAC and monitored HIV-1 replication by virion production using a p24 ELISA. We harvested viruses at the time of peak p24 production and used the aforementioned amounts of viruses to infect fresh Hut78/R5 cells, initiating the next round of spreading infection. Using this method, we propagated NL4-3 and NL4-3-plusAC in three passages. We found that NL4-3 reached peak virus production between days 5 and day 7 in all three passages, except for one passage 2 infections, which peaked at day 9 (Fig. 1B). Whereas NL4-3-plusAC reached peak virus production at day 13 in passage 1, day 5 to day 15 in passage 2, and day 7 in passage 3 (Fig. 1C). Thus, NL4-3-plusAC appeared to be improving its replication kinetics during these three passages, suggesting virus adaptation.

### Development of an NGS-based method to examine U3-R junction sequences using DNA from infected cells

**Fig 2:**
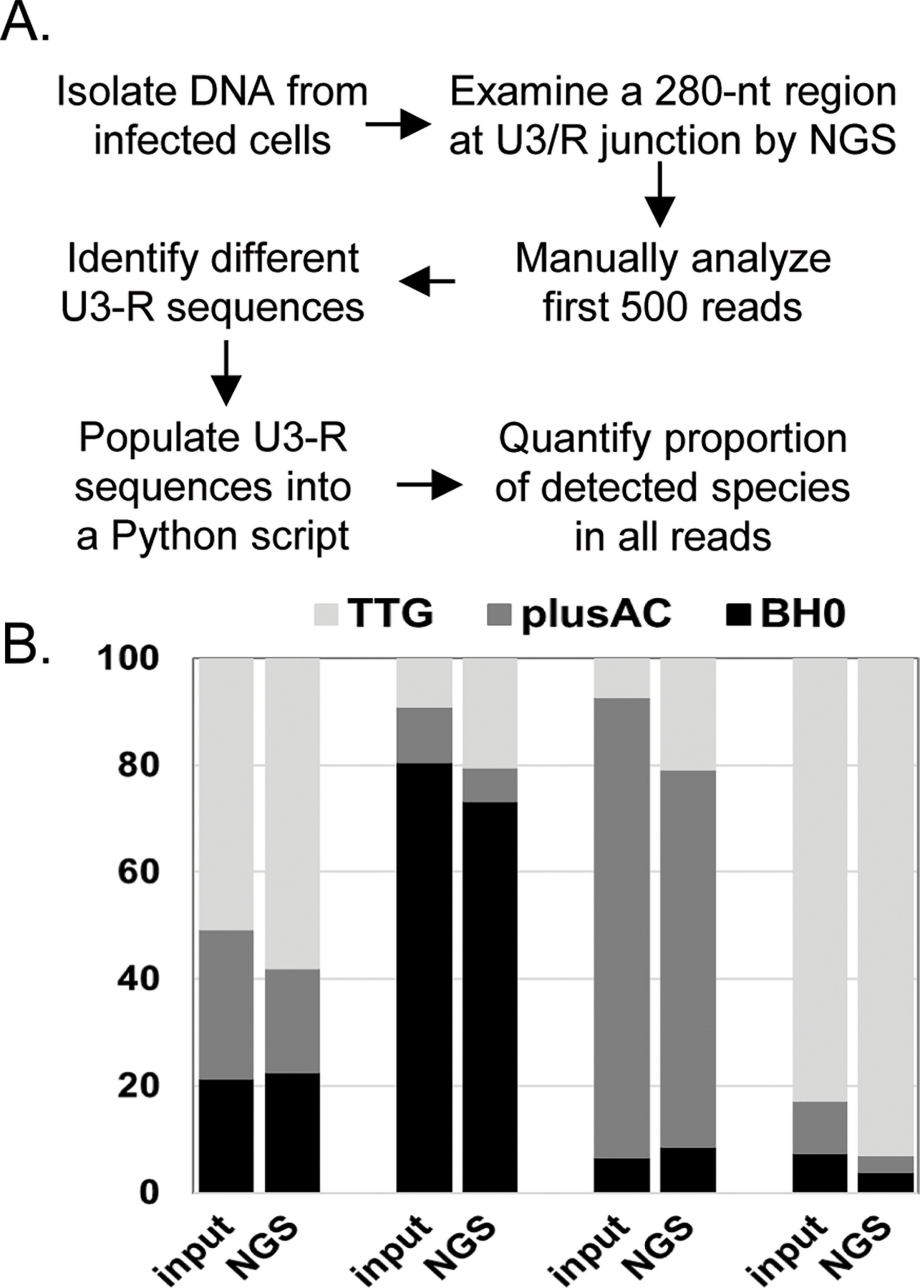
Development of an NGS-based method to analyze U3-R junction sequences. (**A**) Experimental workflow. (**B**) Validation of the NGS-based U3-R assay. Total DNAs were isolated from cells infected with BH0, BH0-TTG (TTG), or BH0-plusAC (plusAC), and the HIV-1 provirus copy numbers were quantified. These DNAs were mixed so that the BH0, TTG, plusAC proviral DNAs were at ratios of 21%, 28%, 51% for mix-1; 81%, 11%, 8% for mix-2; 7%, 86%, 7% for mix-3; and 7%, 10%, 83% for mix-4. The ratios of the mixtures are indicated as input, whereas the results obtained using the NGS U3-R assay are indicated as NGS.

We have previously shown that the region between CATA/TATA box and the three consecutive guanosines at the start of R regulates HIV-1 transcription initiation (12). To better understand the possible changes during virus passaging, we developed a next generation sequencing (NGS) assay to examine sequences near the U3-R junction of proviruses in infected cells. In this assay, a pool of custom NGS primers was used to amplify a region encompassing the U3-R junction from proviral DNA (13). Illumina indexes were added to the amplified products prior to library pooling and sequencing by MiSeq (Fig. 2A). To determine whether the NGS-based U3-R assay could accurately quantify the proportion of different viral species in the cells, we performed the following validation experiment. We isolated DNA from three cell lines, each containing proviruses from an NL4-3-based vector, BH0, BH0-TTG, or BH0-plusAC (12). These vectors contain the *cis*-acting elements required for HIV-1 replication, including intact LTRs, but do not express functional Env. Importantly, these three vectors are identical except that BH0-TTG and BH0-plusAC each has a 2-nt difference in the LTRs compared to BH0. After quantifying the amount of proviruses in DNA isolated from each cell line, we mixed DNAs from the three cell lines at different ratios and sequenced these samples using the NGS U3-R assay. The NGS reads were filtered for quality, converted to FASTA format, and the numbers of BH0, BH0-TTG, and BH0-plusAC reads were determined (Fig. 2A). We found that the new NGS U3-R assay generated results parallel to the expected proportions of different DNA species in the samples (Fig. 2B), thereby validating the assay.

### Detection of different sequences near the U3-R junction during virus passaging

As shown in Fig. 1C, NL4-3-plusAC, but not NL4-3, improved its replication kinetics during serial passaging in Hut78/R5 cells. To determine whether NL4-3 had acquired mutations near the U3-R region during passaging, we isolated DNAs from cells in spreading infections two days prior to peak HIV-1 production and analyzed the proviruses using the NGS U3-R assay. More than 19,000 reads were analyzed for each sample. We first analyzed the wildtype NL4-3 virus and found that, in all 12 samples (three passages, four independent cultures), the vast majority of viruses (>96%) had the original sequence at the U3-R junctions (Table 1). We identified three minor genotypes in which GGG was changed to AGG (≤2%) or GAG (≤0.3%), and a T-to-G substitution immediately upstream of the three consecutive guanosines (≤0.6%). Importantly, these species remained as minor viral populations throughout the three passages (Table 1).

**TABLE 1:** Proportions of U3-R junction sequence genotypes in proviruses from cells infected with WT NL4-3 viruses during serial passaging in Hut78/R5 cells^*a*^

Sequence^*b*^	Sequence name	NL4-3 #A			NL4-3 #B			NL4-3 #C			NL4-3 #D		
		P1	P2	P3	P1	P2	P3	P1	P2	P3	P1	P2	P3
catataagcagctgctttttgcctgtactGGGTCTCTCTGG	GGG	98.1	96.8	96.2	98.4	98.0	96.8	97.9	97.5	0.2	97.9	97.6	96.5
catataagcagctgctttttgcctgtact**A**GGTCTCTCTGG	**A**GG	0.9	1.7	2.0	1.0	1.2	1.9	1.3	1.6	0.1	1.2	1.4	2.0
catataagcagctgctttttgcctgtactG**A**GTCTCTCTGG	G**A**G	0.2	0.3	0.3	0.1	0.2	0.3	0.2	0.3	0.0	0.3	0.2	0.3
catataagcagctgctttttgcctgtac**g**GGGTCTCTCTGG	**g**GGG	0.5	0.6	0.6	0.2	0.2	0.4	0.3	0.3	0.6	0.3	0.4	0.5

^*a*^ The GGG and substitutions in these positions are underlined. Detected changes to NL4-3-WT sequence are in bold.

^*b*^ U3 promoter sequences are shown in lower case, while R region nucleotides are in capital letters.

**Fig 3:**
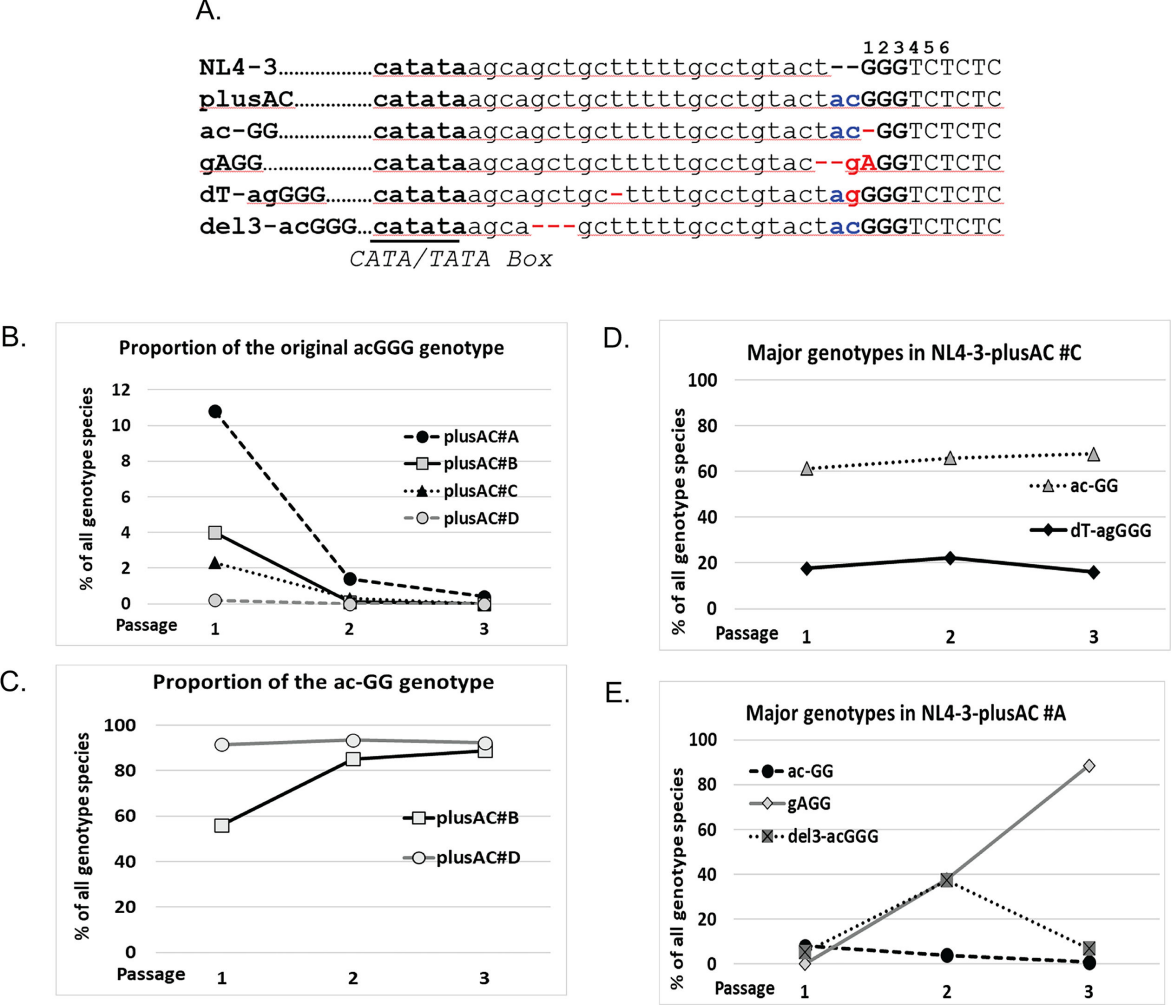
The emergence of NL4-3-plusAC revertants during virus passaging experiments. (**A**) Sequences near the U3-R junction in NL4-3, NL4-3-plusAC, and revertants. Labels and abbreviations are the same as in Fig. 1A. (**B**) Proportions of the NL4-3-plusAC genotype in the four independent cultures. (**C**) Proportions of revertant ac-GG in cultures #B and #D. (**D**) Proportions of revertants ac-GG and dT-agGGG in culture #C. (**E**) Proportions of revertants ac-GG, gAGG, and del3-acGGG in culture #A.

We then examined the U3-R junctions of NL4-3-plusAC proviruses in the serial passaging experiments and found a highly diverse population of genotypes (Table 2 and Fig. 3A). In all four cultures, the NL4-3-plusAC virus rapidly decreased during the passaging experiment (Fig. 3B). NL4-3-plusAC was detected at 11% of the population in one culture and in <5% of the population in the other three cultures in passage 1, in ≤1.4% in all cultures in passage 2, and was barely detectable by passage 3. Of the multiple changes detected, one mutant (ac-GG) was detected in all four cultures; this mutant had a deletion in one of the three guanosines, changing the sequence from acGGG to ac-GG (Fig. 3A). In two cultures (culture #B and #D), ac-GG emerged and became the major genotype in passage 1 and maintained its dominance until the end of the experiment (Fig. 3C). In another culture (culture #C), ac-GG also emerged and became the major virus in passage 1; however, another mutant, dT-agGGG, also emerged, and the two mutants maintained their ratios in all three passages, with ac-GG at ~65% and dT-agGGG at ~20% (Fig. 3D). The mutant dT-agGGG had two alterations compared to NL4-3-plusAC: a 1-nt deletion within five consecutive thymidines and a C-to-G substitution immediately upstream of the GGG sequence (Fig. 3A). NL4-3-plusAC culture #A had a more complex pattern (Fig. 3E); multiple mutants with >5% presence were detected in passage 1, including the ac-GG mutant. In contrast to the other three cultures, ac-GG did not become the dominant virus but decreased with time. Instead, two other mutants, gAGG and del3-acGGG, increased their ratios from passage 1 to passage 2, and gAGG became the dominant virus in passage 3. The revertant del3-acGGG maintained the original acGGG sequence and acquired a 3-nt deletion between the CATA/TATA box and the three consecutive guanosines. In the revertant gAGG, the original acGGG sequence changed to gAGG, which also shortened the distance between the CATA/TATA box and the start of the R region. In contrast to the NL4-3, where the input viral sequence maintained its dominance in all four cultures, NL4-3-plusAC underwent rapid changes in all cultures. Furthermore, all mutants that became dominant contained deletions between the CATA/TATA box and the start of the R region, thereby correcting the AC dinucleotide insertion. These results further support our previous observation that the distance between the CATA/TATA box and the guanosines regulates transcription initiation.

**TABLE 2:** Proportions of U3/R junction genotypes in proviruses from cells infected with mutant NL4-3-plusAC viruses during serial passaging in Hut78/R5 cells

Sequenc^*a*^	Sequence name	NL4-3-plusAC #A			NL4-3-plusAC #B			NL4-3-plusAC #C			NL4-3-plusAC #D		
		P1	P2	P3	P1	P2	P3	P1	P2	P3	P1	P2	P3
catataagcagctgctttttgcctgtactacGGGTCTCTCTGG	acGGG	10.8	1.4	0.4	4	0.1	0	2.3	0.3	0	0.2	0	0
catataagcagctgctttttgcctgtacta**g**GGGTCTCTCTGG	a**g**GGG	12.3	0	0	1.2	0	0	2.9	1	1	0.2	0	0
catataagcagctgctttttgcctgtactacG**A**GTCTCTCTGG	acG**A**G	0.2	0	0	0	0	0	0	0	0	0	0	0
catataagcagctgctttttgcctgtacta**g**G**A**GTCTCTCTGG	a**g**G**A**G	0.2	0	0	0	0	0	0	0	0	0	0	0
catataagcagctgctttttgcctgtactacGG**A**TCTCTCTGG	acGG**A**	24.8	2.3	0.1	20	1.7	0.2	6.5	1.3	0.2	0.2	0	0
catataagcagctgctttttgcctgtacta**g**GG**A**TCTCTCTGG	A**G**GG**A**	1.5	0	0	0.3	0	0	0.1	0	0	0	0	0
catataagcagctgctttttgcctgtactac **T**GGTCTCTCTGG	ac**T**GG	5	0.2	0	8	1.5	0.1	0	0	0	0.2	0	0
catataagcagctgctttttgcctgtactacGG**T**TCTCTCTGG	acGG**T**	0.1	0	0	0.3	0	0	0.2	0	0	0	0	0
catataagcagctgctttttgcctgtacta**a**GGGTCTCTCTGG	a**a**GGG	0.4	0	0	0.1	0	0	0.1	0	0	0	0	0
catataagcagctgctttttgcctgtactac- GGTCTCTCTGG	ac-GG	8	3.9	0.8	56.1	85.2	88.8	61.4	65.9	67.7	91.5	93.5	92.3
catataagcagctgctttttgcctgtactac- **T**GTCTCTCTGG	ac-**T**G	0.1	4.9	1.1	0.2	0.4	0.2	0	0	0	0	0.1	0
catataagcagctgctttttgcctgtactac- G**A**TCTCTCTGG	ac-G**A**	0.2	0	0	1	0.2	0.1	0.3	0.2	0.1	0.1	0.1	0
catataagcagctgctttttgcctgtactac-G**T**TCTCTCTGG	ac-G**T**	0	0	0	0.2	0.3	0.5	0.2	0.4	0.6	0.2	0.1	0.2
catataagcagctgctttttgcctgtacta- GGGTCTCTCTGG	a-GGG	0.4	0.6	0	2.4	4.5	4	3.7	2.8	2.3	3.7	3.8	3.6
catataagcagctgctttttgcctgtacta- **A**GGTCTCTCTGG	a-**A**GG	0	0	0	0.1	0.1	0.2	0.1	0.1	0.1	0.2	0.1	0.1
catataagcagctgctttttgcctgtacta- GG**A**TCTCTCTGG	a-GG**A**	0.1	0	0	0.2	0	0	0.1	0	0	0	0	0
catataagcagctgctttttgcctgtacta- **T**GGTCTCTCTGG	a-**T**GG	0.1	0	0	0.5	0.6	0.1	0.1	0	0	0.1	0.1	0
catataagcagctgctttttgcctgtac**g**a- GGGTCTCTCTGG	**g**a-GGG	0	0	0	0	0	0	0.1	0.1	0.3	0	0	0.1
catataagcagctgctttttgcctgtac**g**ac- GGTCTCTCTGG	**g**ac-GG	0.3	0.5	0.2	0.4	0.4	1	1.1	1.5	2	1.2	1.5	2.5
catataagcagctgctttttgcctgtac**a**ac- GGTCTCTCTGG	**a**ac-GG	0.5	0.2	0	3.2	4.1	0	0.1	0.1	0.4	0	0	0.1
*catataagcagctgctttttgcctgtact--GGGTCTCTCTGG*	*GGG* ^*b*^	*0.1*	*0.1*	*0.1*	*0*	*0.1*	*0*	*0.1*	*0.2*	*0.1*	*0.1*	*0.1*	*0.2*
catataagcagctgctttttgcctgtac**g**--GGGTCTCTCTGG	**g**GGG	0	0.2	0.6	0	0	0	0	0	0.1	0	0	0
catataagcagctgctttttgcctgtac**g**--**A**GGTCTCTCTGG	**gA**GG	0	37.8	88.5	0	0	0	0	0	0	0	0	0
catataagcagctgctttttgcctgtac**g**acGG**A**TCTCTCTGG	**g**acGG**A**	24.9	8.5	0.8	0.6	0	0	0.4	0.2	0	0	0	0
catataagcagctgctttttgcctgtac**g**acGGGTCTCTCTGG	**g**acGGG	1.6	0.9	0.1	0.1	0	0	0.1	0.1	0	0	0	0
catataagcagctgctttttgcctgtac**g**a**g**GGGTCTCTCTGG	**g**a**g**GGG	0.4	0	0	0	0	0	0.1	0	0	0	0	0
catataagcagctgctttttgcctgtact**gg**GGGTCTCTCTGG	**gg**GGG	0.3	0	0	0	0	0	0.1	0	0	0	0	0
catataagcagctgctttttgcctgtact**gc**GGGTCTCTCTGG	**gc**GGG	0.1	0	0	0.1	0	0	0	0	0	0	0	0
catataagcagctgc-ttttgcctgtactacGGGTCTCTCTGG	dT-acGGG	0.4	0	0	0.1	0	0	0.7	0.2	0	0	0	0
catataagcagctgc-ttttgcctgtacta**g**GGGTCTCTCTGG	dT-a**g**GGG	0.8	0	0	0	0	0	17.6	22.2	16	0	0	0
catataagcagctgc-ttttgcctgtacta**g**GG**A**TCTCTCTGG	dT-a**g**GG**A**	0.1	0	0	0	0	0	0.3	0.2	0	0	0	0
catataagcagctgc-ttttgcctgtacta**g**GG**T**TCTCTCTGG	dT-a**g**GG**T**	0	0	0	0	0	0	0.1	0.1	0.1	0	0	0
catataagcagctgc-ttttgcctgtactac- GGTCTCTCTGG	dT-ac-GG	0	0	0	0	0.1	0.4	0.8	1.4	2.1	0.1	0.2	0.3
catataagcagctgc-ttttgcctgtacta**g**CGGTCTCTCTGG	dT-a**gC**GG	0	0	0	0	0	0	0	0.3	0.8	0	0	0
catataagcagctgc-ttttgcctgtacta**a****C**GGTCTCTCTGG	dT-a**ac**GG	0	0	0	0	0	0	0.2	0.6	1.8	0	0	0
catataagcagctgc-ttttgcctgtacta**-** GGGTCTCTCTGG	dT-a-GGG	0	0	0	0	0	4	0	0	1.9	0	0	0
catataagcagctgc-ttttgcctgtacta**t****C**GGTCTCTCTGG	dT-a**tC**GG	0	0	0	0	0	0	0	0	0.2	0	0	0
catataagcagctgc-ttttgcctgtacta**a**GGGTCTCTCTGG	dT-a**a**GGG	0	0	0	0	0	0	0	0	0.4	0	0	0
catataagca---gctttttgcctgtactacGGGTCTCTCTGG	del3-acGGG	5.2	37.3	6.9	0	0	0	0	0	0	0	0	0
catataagca---gctttttgcctgtactacGG**A**TCTCTCTGG	del3-acGG**A**	0.5	0.7	0	0	0	0	0	0	0	0	0	0
catataagcagctgctttttgcctgtact-**g**GGGTCTCTCTGG	t-**g**GGG	0	0.1	0	0	0.1	0.2	0.1	0.1	0.1	0	0.1	0.2
catataagcagctgctttttgcctgtac**aaa**GGGTCTCTCTGG	**aaa**GGG	0	0	0	0.2	0	0	0	0	0	1.4	0.1	0
catataagcagctgctttttgcctgtac-**aa**GGGTCTCTCTGG	**c-aa**GGG	0	0.1	0	0.2	0.2	0	0	0	0	0	0	0

^*a*^ U3 promoter sequences are shown in lower case whereas R region nucleotides are in capital letters. The GGG and substitutions in these positions are underlined; detected changes to NL4-3-plusAC sequence are in bold.

^*b*^ Italics indicate genotype corresponding to the wildtype NL4-3 sequence. P1, P2, and P3 refers to passage 1, 2, and 3, respectively.

### Characterization of the NL4-3-plusAC adaptive mutants

**Fig 4:**
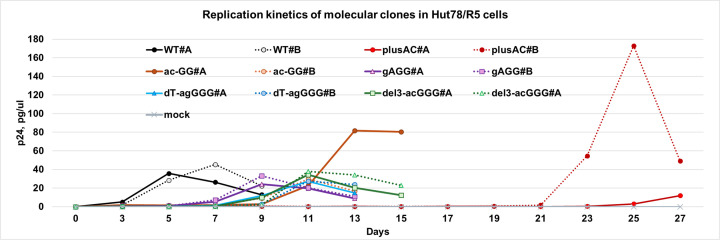
Replication kinetics of NL4-3, NL4-3-plusAC, and its revertants in Hut78/R5 cells.

We observed four NL4-3-plusAC adaptive mutants that became dominant viral species in the passaging experiments: ac-GG, gAGG, dT-agGGG, and del3-acGGG. To determine whether the mutations between the CATA/TATA box and the beginning of the R region improve the replication of these viral species, we generated four NL4-3-based mutant molecular clones containing these modifications in both LTRs. We then produced viruses, infected Hut78/R5 cells, and monitored HIV-1 replication kinetics by virus production using p24 ELISA (Fig. 4). In these experiments, we observed that the virus production of all four adapted mutants peaked between day 9 to day 13, which is an improvement over NL4-3-plusAC, which peaked after day 25 in these experiments. Compared to the results shown in Fig. 1, the NL4-3-plusAC virus peaked at later times in these experiments, probably because we used a different cell stock with cells that were less susceptible to HIV-1 infection.

**Fig 5:**
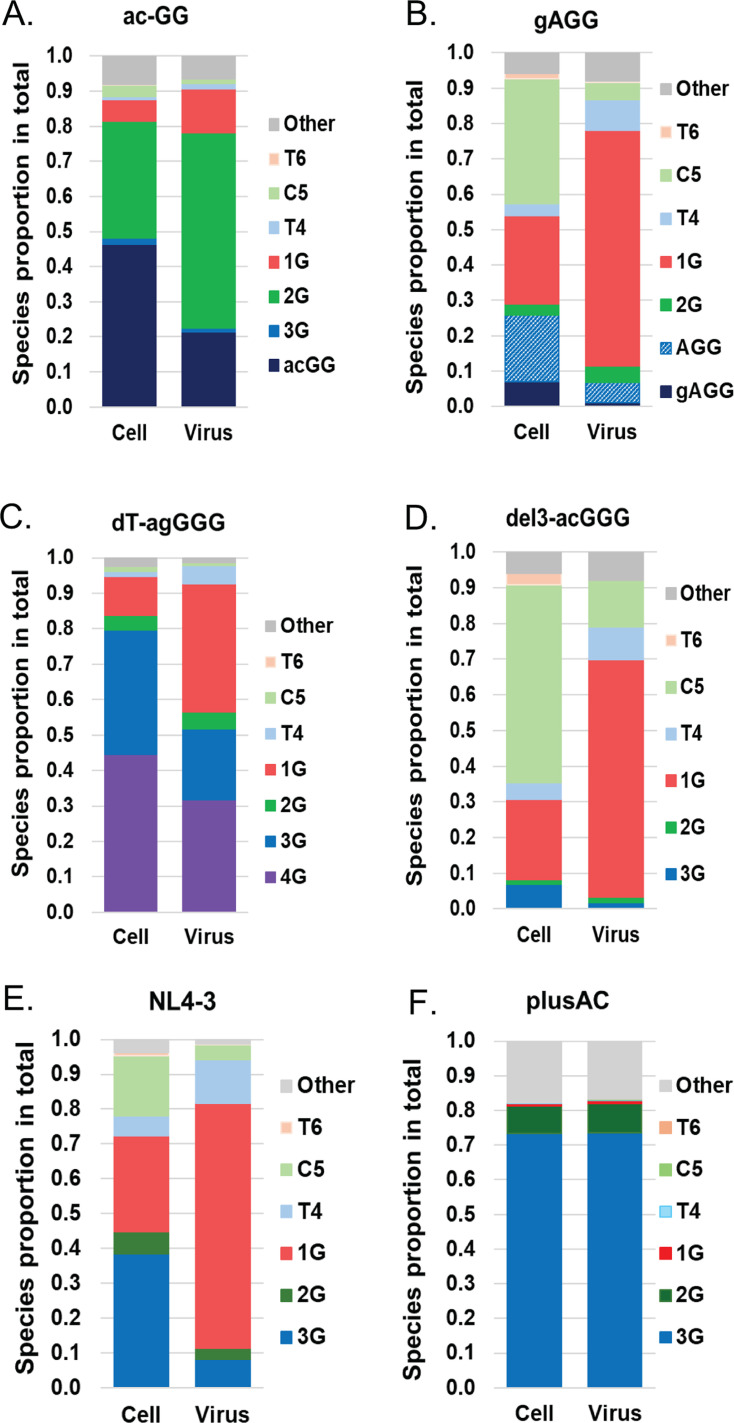
Proportion of RNA species produced and packaged by NL4-3-plusAC revertants determined by 5′ RACE-NGS method. Unspliced RNA species expressed by proviruses in infected cells (cell) and packaged into particles (virus) by revertant NL4-3-ac-GG (**A**), NL4-3-gAGG (**B**), NL4-3-dT-agGGG (**C**), and NL4-3-del3-acGGG (**D**), wildtype NL4-3 (**E**), and mutant NL4-3-plusAC (**F**). A to D, data shown are combined reads from two independent NGS experiments. Previously published data (12) (**E and F**) are shown for comparison.

To better understand how these mutations changed HIV-1 transcription initiation and whether there are preferences in genome packaging, we analyzed the 5′ ends of cellular HIV-1 RNA and virion RNA from particles produced by infected cells using the previously described NGS-based 5′ RACE (12). In the NL4-3-ac-GG virus, which was detected in all cultures, transcription mostly initiated at the A and at the first G of the ac-GG to generate acGG RNA and 2G RNA, respectively, along with some 1G RNA. Of these RNAs, 1G and 2G RNA were enriched in the virions (Fig. 5A). In the NL4-3-gAGG mutant, which became the major virus in one culture, three major transcripts were detected. These were initiated from the A and the last G of gAGG, generating AGG RNA and 1G RNA, respectively, and the cytosine located at position 5 (shown in Fig. 1A and 3A) generating the C5 RNA (Fig. 5B). Of these RNAs, 1G RNA is enriched in virions (Fig. 5B). In the NL4-3-dT-agGGG virus, transcription mostly initiated from the first, the second, and the last guanosines of agGGG, generating 4G, 3G and 1G RNA (Fig. 5C). Of these RNAs, 1G RNA is enriched in the virions (Fig. 5C). In the NL4-3-del3-acGGG virus, transcription mostly initiated at the last G of the acGGG and the cytosine at position 5, generating 1G RNA and C5 RNA (Fig. 5D). Of these RNAs, 1G RNA is enriched in the virions. The 2G RNA is expressed in all four revertants; however, only NL4-3-ac-GG exhibits virion enrichment of 2G RNA. Each of these revertants has a different mixture of transcripts. It is likely that, in the case of NL4-3-ac-GG, the 2G RNA is more favorably packaged than the acGG RNA, and the 1G RNA expression is very low. The combination of these two factors allows for the enrichment of 2G RNA.

We have previously shown that NL4-3 expresses multiple transcripts, including 3G and 1G RNA, in the cells and preferentially packages 1G RNA (Fig. 5E). In contrast, NL4-3-plusAC mainly expresses 3G RNA and packages 3G RNA (Fig. 5F). In all four adapted mutants, there are more than one major transcript, which is a shift from a mutant phenotype with one major transcript (NL4-3-plusAC) (Fig. 5F) to a wildtype-like phenotype with multiple major transcripts (NL4-3) (Fig. 5E). Importantly, all four mutants substantially increased the transcription of 1G RNA compared to the plusAC mutant, and the 1G RNA was preferentially packaged, which is also a shift from an NL4-3-plusAC phenotype to an NL4-3 phenotype. Therefore, although these mutants have distinct changes at the U3-R junctions and produced different transcripts, they all yielded phenotypes more similar to NL4-3 than to NL4-3-plusAC.

### Rate of copying the guanosine cap during one round of HIV-1 replication

**Fig 6:**
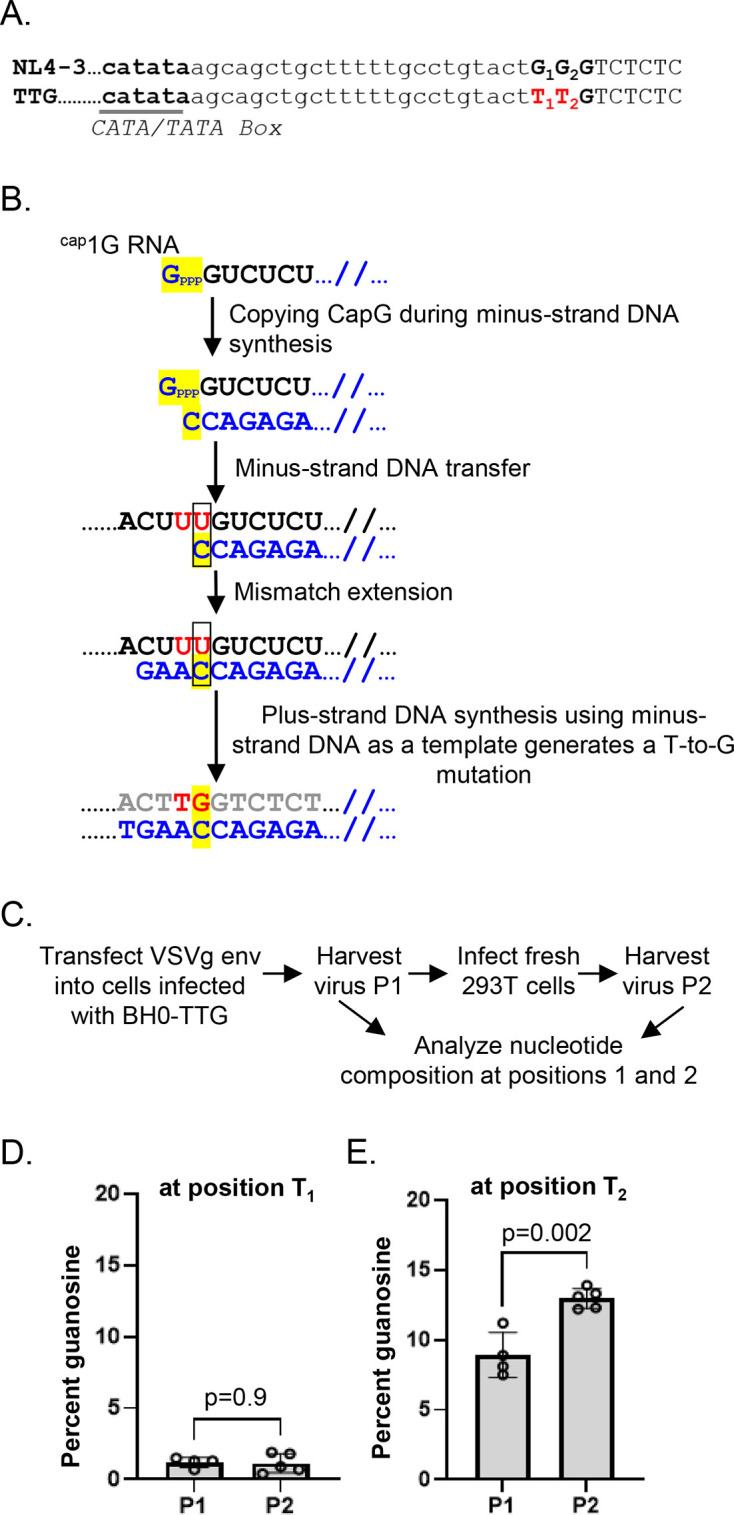
Substitution rate caused by copying the guanosine cap in one round of HIV-1 replication. (**A**) Sequences near the U3-R junction in NL4-3 and the TTG mutant. Labels and abbreviations are the same as in Fig. 1A. Two nucleotide substitutions in the TTG mutant are shown in red. (**B**) The proposed mechanism for frequent mutation adjacent to the transcription start site. The example shown is T-to-G reversion at the T2 position of NL4-3-TTG during reverse transcription. RNA sequences are shown in black, with the two mutated nucleotides of the TTG mutant shown in red; The guanosine cap (Gppp) and its derived sequences are highlighted in yellow. Mismatched base pairs are indicated by boxes. Minus- and plus-strand DNA sequences are shown in blue and gray, respectively. (**C**) Experimental workflow for measuring T-to-G substitutions during one round of HIV-1 replication from passage 1 (P1) to passage 2 (P2). The percentage of guanosine detected in the first nt of R, position T_1_ (**D**) and the second nt of R, position T_2_ (**E**) in BH0-TTG during one round of HIV-1 replication. *P* values were obtained by one-way ANOVA.

In both NL4-3 and NL4-3-plusAC, we detected sequences in which the nucleotide preceding transcription initiation was converted to guanosine. For example, in NL4-3, the nucleotide before GGG is a T, and we observed T-to-G mutations that changed tGGG to gGGG (Table 1), and in NL4-3-plusAC, we observed acGGG-to-agGGG mutations (Table 2). Similarly, we have previously reported that during serial passaging in spreading infection, the mutant NL4-3-TTG, in which the three consecutive guanosines were changed to TTG (Fig. 6A), quickly reverted the second thymidine to the wildtype guanosine while maintaining the first thymidine (TTG to TGG). NL4-3-TTG predominantly generates and packages 1G RNAs. We hypothesize that reverse transcriptase (RT) copies the guanosine cap of the 1G RNA during DNA synthesis, resulting in the rapid T-to-G reversion (Fig. 6B; [12]). The observation of similar mutants in NL4-3 and NL4-3-plusAC provides further evidence that the guanosine cap can sometimes be copied during DNA synthesis and introduce mutations during HIV-1 replication. However, the frequency of such substitution events is unknown; both the previous study and the current results were derived from multiple rounds of HIV-1 replication, in which selection pressure plays a major role in the emergence of mutants.

To better quantify the rate at which copying the guanosine cap alters the viral genome sequence, we measured its frequency in one round of HIV-1 replication. For this purpose, we used previously described BH0-TTG, an NL4-3-based vector that expressed functional Gag/Gag-Pol, Tat, Rev, and a surface marker, HSA, in the *nef* reading frame. Additionally, the three consecutive guanosines in both LTRs were changed to TTG (12). BH0-TTG does not express functional Env and cannot spread in cells by itself; however, when supplemented with HIV-1 Env or G protein from vesicular stomatitis virus (VSV-G), it can undergo one round of replication. We generated a cell line infected with BH0-TTG at low multiplicity of infection (MOI) and enriched infected cells by cell sorting using the HSA marker (12). We then mobilized the BH0-TTG by transfecting the cell line with plasmid expressing VSV-G, harvested viruses (designated as P1 virus), and infected fresh cells; the BH0-TTG provirus in the newly infected cells can express viral RNA and proteins to produce HIV-1 particles (designated as P2 virus). By comparing the P1 and the P2 virus, we can determine the rate at which cap copying alters viral sequences (Fig. 6C). We measured the sequence alterations of P1 and P2 viruses using a previously described RT-PCR sequencing method (14). We found that the proportion of guanosine at position 1 remained low and unchanged between P1 and P2 virus (1.3 ± 0.5% and 1.2 ± 0.7%, respectively, *P* = 0.9; one-way ANOVA; Fig. 6D), whereas the proportion of guanosine at position 2 increased from 8.9 ± 1.6 to 13.0 ± 0.7 between P1 and P2 virus, respectively (*P* = 0.002; one-way ANOVA; Fig. 6E). Considering that 1G RNA represents 78% of all packaged gRNA in BH0-TTG virions (12), these data indicate that the T-to-G substitution occurred in ~5% (4% /0.78) of events in one round of replication. This rate is at least ~1,000 fold higher than the general substitution rate elsewhere in the viral genome in one round of replication (15, 16). The difference in genetic stability between position 1 and position 2 further supports the hypothesis that the T-to-G substitution is the result of copying of guanosine cap during reverse transcription.

## DISCUSSION

HIV-1 generates multiple unspliced transcripts that vary by a few nt at the 5′ end but differ functionally. Most HIV-1 expresses 3G RNA and 1G RNA as two major transcripts. Mutants that mostly express one major transcript, either 1G RNA or 3G RNA, have replication fitness defects (12). Here, we propagated and passaged the NL4-3-plusAC mutant virus, which has an AC dinucleotide insertion upstream of the three consecutive guanosines and mostly expresses 3G RNA (12). We found that multiple revertants with distinct genotypes quickly arose and the adapted revertants have shortened distances between the CATA/TATA box and the start of the R region compared to NL4-3-plusAC. Molecular clones containing these revertant mutations regained the ability to express more than one major transcript and selectively package 1G RNA. Furthermore, the revertants have improved replication kinetics compared with the NL4-3-plusAC virus. Our findings indicate that the ability to express two functionally distinct unspliced RNAs is necessary for optimal HIV-1 replication fitness. We hypothesized that the NL4-3-plusAC can use different paths to regain multiple transcripts: it can restore the same 1G and 3G RNA balance like the wildtype NL4-3, or it can use new RNA species. In four major revertants we isolated, only one revertant expressed significant proportions of both 1G and 3G RNA (dT-agGGG). Furthermore, all four revertants had major transcripts that are neither 1G nor 3G, including ac-GG RNA (Fig. 5A), AGG RNA (Fig. 5B), 4G RNA (Fig. 5C), and C5 RNA (Fig. 5B and D). Importantly, in all four mutants, the expression of 1G is recovered and it is preferentially packaged (Fig. 5). In contrast, the expression of 3G RNA is less obligatory; only one of four revertants (dT-agGGG) retains 3G RNA as a major RNA species. These results indicate that the function of the packageable RNA (1G RNA) is difficult to replace by other RNA species; therefore, all major revertants need to find a path to express 1G RNA. In contrast, the function of the translation template is more easily replaced, hence many revertants use other RNA species to replace the 3G RNA functions. Taken together, these findings illustrate the importance of expressing different RNA species to serve distinct functions. Of the two major functions of HIV-1 RNA, there is more selection pressure for the RNA packaging substrate compared to the translation template because multiple RNA species can at least partially replace 3G RNA functions.

**Fig 7:**
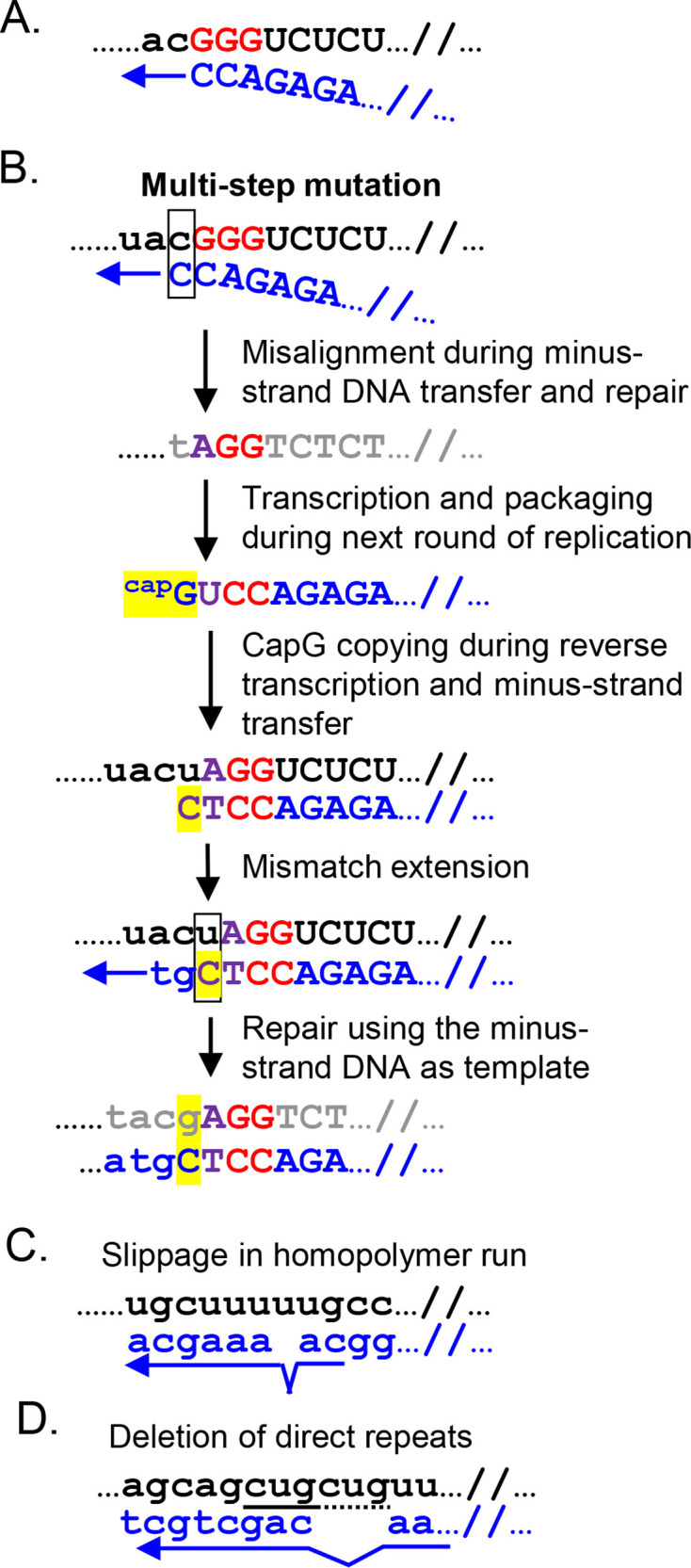
Proposed pathways for generating different revertants of NL4-3-plusAC mutant. (**A**) Generation of ac-GG revertant by misalignment during minus-strand DNA transfer. (B) Generation of gAGG revertant by G-to-A mutation and copying of capG during reverse transcription. (**C**) Deletion of one thymidine in a polyT stretch between CATA/TATA box and GGG motif. (**D**) Generation of del3-acGGG revertant by deletion of direct CTG repeat in the U3 sequence (underlined with solid and dashed lines). Viral RNA is in black; minus- and plus-strand DNAs are in blue and grey, respectively. GGG at the start of R are in red, and the G-to-A mutation is in purple. CapG and its derivatives are highlighted in yellow with the mismatch region boxed.

Using an NGS-based assay, we have shown that the sequences near the U3-R junctions experience frequent mutations. In the wildtype NL4-3, we mostly observe three changes (Table 1), two of which are G-to-A substitutions, possibly caused by error-prone reverse transcriptase, as previously described (15–17). The other is a T-to-G substitution directly upstream of the three guanosines, which likely occurred through copying of the guanosine cap of the 3G RNA (12). In contrast, we observed a large array of changes while propagating the NL4-3-plusAC virus (Table 2). In addition to mutations described above, the G-to-A substitution and the guanosine substitution upstream of the transcription start sites, there are multiple mutants with 1- to 3-nt deletions. In four independent NL4-3-plusAC cultures, we observed four revertants that emerged to be a major species in passage 2 and/or passage 3. The most frequent mutant we observed is an acGGG to ac-GG mutation. The ac-GG mutant was observed in all four cultures and became a dominant species in three cultures, indicating that it occurs relatively frequently and has a selective advantage over NL4-3-plusAC and many other mutants. We propose that the deletion results from misalignment of minus-strand DNA transfer. The plusAC virus expresses and packages 2G RNA at a low frequency (~7%, Fig. 5F) (12). It is possible that during minus-strand DNA transfer, a misalignment occurred in which the nascent CC dinucleotide aligned with the first two Gs instead of the second and third guanosines (Fig. 7A), resulting in the loss of a guanosine. The gAGG revertant was observed in only one of the four cultures and has an acGGG to gAGG change. The generation of the gAGG revertant is likely more complex; there are multiple scenarios by which this mutant can arise, most of which involve more than one step. For example, it is possible that similar to the mechanism described in Fig. 7A, misalignment occurred during minus-strand DNA transfer, resulting in the loss of the CG dinucleotide and the generation of a revertant with a tAGG sequence at the U3-R junction (Fig. 7B). This intermediate revertant could generate and package an RNA species with an AGG sequence at the 5′ end. During further replication, the guanosine cap was copied, resulting in a T-to-G substitution and generating the gAGG genotype. (Fig. 7B).

Besides revertants generated by possible minus-strand DNA transfer errors, we observed two other deletion mutants between the CATA/TATA box and the three guanosines. One is a T deletion within a run of five Ts (Fig. 7C). Reverse transcriptase is known to slip while copying homopolymers (15, 18). Furthermore, a T deletion mutation was observed in two clinical isolates (10). The other mutant has a 3-nt deletion from AGCTGCT to AGCT, losing one of the two GCT repeats (Fig. 7D). Deletion of short direct repeats is known to occur during reverse transcription when RT dissociates from the template and reassociates with the other copy of the repeated sequence in the template (15, 18). Although both deletions are known to occur during reverse transcription, we did not observe these two deletions in NL4-3 passages. It is likely that these deletion mutants were enriched during NL4-3-plusAC propagation due to their improved replication fitness compared to the original NL4-3-plusAC virus. In contrast, such deletions in the NL4-3 virus might not provide a selective advantage and were not enriched. Although many substitution mutations were observed in the plusAC mutant and in wildtype NL4-3 virus (Tables 1 and 2), these mutants did not become dominant in the culture, likely because they do not have a strong replication advantage. Interestingly, during the passage of the plusAC virus passage, we detected the AC dinucleotide deletion at very low frequencies (<0.2%) in some cultures (Table 2); this deletion reverts the sequence back to the wildtype NL4-3 genotype. However, this revertant did not become dominant. We reasoned that the 2-nt deletion occurred infrequently and likely arose late in the culture, thereby not having enough time to enrich in the culture. Additionally, very small aliquots of the viruses were used to infect cells to start the next passage; this protocol may not transfer all minor species in the culture; thus, the dinucleotide deletion may need to occur *de novo* in each passage, reducing its chance to emerge in the culture.

In the passaging of NL4-3 and NL4-3-plusAC viruses (Tables 1 and 2), as well as the previously reported TTG virus (12), we observed that G substitution occurred immediately upstream of the major transcription start sites. This substitution is particularly striking in the TTG virus, where the T immediately upstream, but not the T 2-nt upstream of the G, reverted rapidly (12). We have hypothesized that the substitution occurs not by mis-incorporation of nucleotides during DNA synthesis but by RT copying the guanosine cap, resulting in a mismatch between the nascent DNA and RNA template after minus-strand DNA transfer; RT then extends the mismatched sequence to continue DNA synthesis. During plus-strand DNA synthesis, the minus-strand DNA is used as a template, resulting in the G substitution. Here, we have measured the frequency of this reversion in one round of replication to be 5%, which includes the rate at which RT copies the guanosine cap and the rate at which mismatch extension occurs during minus-strand DNA transfer. As the rate of mismatch extension at this position is not known, the rate at which HIV-1 RT copies the cap during minus-strand DNA synthesis is ≥5%. Importantly, the effect of RT copying the guanosine cap is also observed in samples from people living with HIV-1. We have analyzed the subtype B 5′ LTR sequences (*N* = 357) in the Los Alamos National Laboratory HIV Database (https://www.hiv.lanl.gov/) and examined the sequences near the three consecutive guanosines: T_-6_G_-5_T_-4_A_-3_C_-2_T_-1_**G_1_G_2_G_3_**T_4_C_5_T_6_C_7_T_8_. Of these, only the T in the −1 position (directly upstream of the GGG) has a 1.7% T-to-G substitution. The other five Ts are conserved and do not exhibit polymorphisms, including the T-to-G substitution. Additionally, C_-2_ was also conserved. These results extend our observation to human samples and show that copying of the guanosine cap by RT impacts HIV-1 replication in people. The T_-1_ polymorphism is caused by reverse transcription of 3G RNA. Most HIV-1 particles have 1G RNA and not 3G RNA; thus, by encoding three consecutive guanosines, HIV-1 has the built-in mechanism to absorb this frequent mutation and maintain genetic stability.

## MATERIALS AND METHODS

### Molecular cloning of HIV-1 constructs

The full-length infectious molecular clone pNL4-3 (19), a generous gift from Malcom Martin (NIAID), was obtained through NIH HIV Reagent Program. Two NL4-3 mutants, NL4-3-TTG and NL4-3-plusAC, have been previously described; these constructs contain modifications near the junction of the U3 and R regions in both the 5′ and 3′ LTRs (12). Additional NL4-3-based mutants were generated by replacing the MluI-to-BssHII (5′ LTR) and XhoI-to-NgoMIV (3′ LTR) fragments of NL4-3 with synthesized DNA fragments containing mutations (Integrated DNA Technology, Inc.). The previously described NL4-3-based vector BH0 contains all the *cis*-acting elements required for HIV-1 replication and expresses functional Gag/Gag-Pol, Tat, Rev, and a surface marker HSA in the *nef* reading frame (20). Two previously described BH0 derivatives, BH0-TTG and BH0-plusAC, are identical to BH0 except that each contains mutations in both LTRs (12). BH0, BH0-TTG, and BH0-plusAC do not express Env and can undergo one round of replication when supplemented with the VSV-G envelope.

Plasmid construction was performed using the NEBuilder Gibson Assembly kit (NEB) or standard molecular cloning techniques. All modified regions were verified by Sanger sequencing or nanopore whole-genome sequencing.

### Cell culture, cell lines, and virus infections

Human embryonic kidney 293T cells (from American Type Culture Collection, #CRL-3216) were maintained in Dulbecco’s modified Eagle’s medium supplemented with 10% fetal bovine serum (FBS), 100 U/mL penicillin, and 100 µg/mL streptomycin. Hut78/R5, a derivative of the human T-cell line Hut78 that stably expresses CCR5 (21), was maintained in Roswell Park Memorial Institute Medium (RPMI −1640) supplemented with 10% FBS, 100 U/mL penicillin, and 100 µg/mL streptomycin. All cells were maintained in humidified 37°C incubators with 5% CO_2_. 293T-based cell lines BH0, BH0-TTG, and BH0-plusAC, each containing one provirus per cell, were described previously (12).

To generate virus from NL4-3 or its mutants, 293T cells were transfected with viral constructs using TransIT-LT1 (Mirus Bio). Virus supernatants were collected 24 hours post-transfection, clarified through 0.45-µm-pore-size filters, and quantified by p24 capsid ELISA (XpressBio). To monitor replication kinetics, 25 ng of wildtype NL4-3 or 50 ng of NL4-3-plusAC was used to infect 1 × 10^6^ Hut78/R5 cells; the input virus was removed after 6 hours and replaced with 3 mL of fresh medium. Starting three days post-infection, the culture was split every other day as follows: 2 mL of culture was harvested, centrifuged at 16,000 × g for 10 min, and the cell pellets and supernatant were frozen and stored at −80°C for future analysis. The remaining 1 mL of infected culture was supplemented with 2 mL of fresh medium for continuous culture. The amount of virus in culture supernatant was measured by p24 ELISA (XpressBio).

### RNA and DNA isolation and analysis

Virion RNA was extracted from culture supernatants using a Viral Mini RNA kit (Qiagen). In spreading infections, virion RNA was extracted from samples corresponding to the peak virus production. Cell pellets harvested two days prior to the p24 peak were used to isolate total cellular RNA or total cellular DNA using the Qiagen Mini RNeasy Plus kit or Qiagen DNA Blood Mini kit, respectively.

To determine the frequency of HIV-1 capG copying by reverse transcription, the previously described cell line, in which most cells contain one BH0-TTG provirus (12), was transfected with a VSV-G-expressing plasmid pHCMV-G (22) using TransIT-LT1 reagent (Mirus Bio). Infectious virus stocks (designated P1) were collected 24 hours post-transfection, filtered through a 0.45 µm filter, and used to infect fresh 293T cells. Viruses produced by infected cells (designated P2) were harvested 3 days post-infection, and virion RNAs were isolated from P1 and P2 viruses using a Viral Mini RNA kit (Qiagen). RNA samples were treated with TURBO DNA-free Kit (Invitrogen), and a fragment containing the nef-to-R sequence was amplified using SuperScript II One-Step RT-PCR System with Platinum Taq (ThermoFisher), as previously described (13), followed by Sanger sequencing of purified PCR products. The relative proportions of the wildtype and mutant sequences were calculated by the height of the mutant peak in the chromatogram divided by the sum of the heights of the mutant and the wildtype peaks. Experimental measurements were referenced against the standard curve, which was generated by mixing the wildtype and mutant plasmids at known ratios.

The copy number of proviruses in total cellular DNA was measured by real-time qPCR using gag-specific primers: Spe-WT-F1495 (5′-ATAGCAGGAACTACTAGTACC-3′), Spe-R1564 (5′-CTACTGGGATAGGTGGATTA-3′), and LightCycler 480 SYBR qPCR Kit (Roche).

### NGS-based methods and analysis

A previously described NGS-based 5′ RACE was used to determine the 5′ ends of HIV-1 unspliced transcripts both in infected cells and in virions (12). Briefly, RNA was converted into cDNA using the SMARTer RACE 5′/3′ kit (Takara), followed by PCR with a pool of primers containing Illumina overhang adaptors to amplify the 5′ RACE product prior to sequencing. More than 50,000 reads were analyzed for each sample.

To determine sequences near the U3 and R junction, total cellular DNA was isolated from infected cells. A fragment including the nef-to-R sequence was amplified using a pool of primers that contain a segment that anneals to the HIV-1 genome, a stretch of randomized nucleotides, and Illumina adapter sequences (Table 3) (13). PCR products with Illumina adapters were gel-purified using NucleoSpin Gel and PCR Clean-up Mini kit (Macherey-Nagel), quantified using Qubit dsDNA HS (High Sensitivity) Assay Kit (ThermoFisher), and indexed using Nextera XT Index kit v2 Set B (Illumina) for multiplexing. Libraries were gel-purified and quantified as above, then pooled for sequencing using MiSeq Reagent Kit v2 (300 cycles) (Illumina). More than 19,000 reads were analyzed for each sample.

**TABLE 3:** NGS primers for amplification of proviral U3-R junction sequence

Primer name	Primer sequence^*a*^
NL9301S-NGS-F1	5′-**TCGTCGGCAGCGTCAGATGTGTATAAGAGACAG**GAATGGATGACCCTGAGAG −3′
NL9301S-NGS-F2	5′-**TCGTCGGCAGCGTCAGATGTGTATAAGAGACAG**NNNNNNNTGAATGGATGACCCTGAGAG-3′
NL9301S-NGS-F3	5′-**TCGTCGGCAGCGTCAGATGTGTATAAGAGACAG**NNNNNNNNNNNNNNGAATGGATGACCCTGAGAG-3′
NL9584A-NGS-R1	5′-**GTCTCGTGGGCTCGGAGATGTGTATAAGAGACAG**NNNTTCCCTAGTTAGCCAGAGAG-3′
NL9584A-NGS-R2	5′-**GTCTCGTGGGCTCGGAGATGTGTATAAGAGACAG**NNNNNNNNNNTTCCCTAGTTAGCCAGAGAG-3′
NL9584A-NGS-R3	5′-**GTCTCGTGGGCTCGGAGATGTGTATAAGAGACAG**NNNNNNNNNNNNNTTCCCTAGTTAGCCAGAGAG-3′

^*a*^ NGS adaptor PCR primers consist of three parts: gene-specific region (underlined), variable linker (N), and overhang adaptor sequence for Illumina sequencing (boldface).

### Bioinformatics and statistical analysis

MiSeq NGS reads were analyzed using a custom bioinformatics pipeline. Briefly, after trimming adaptors, NGS reads were filtered for quality (q20q90) and converted into FASTA files. The first 500 reads were examined manually to determine the most frequent sequence species, and the custom Python script was modified to count the identified sequence species in all reads. One-way ANOVA was performed in GraphPad Prism v9.2.0 (GraphPad Software, LLC).

## Data Availability

The NGS results (as .fasta files) are available at https://fsabcl-bioi02p.ncifcrf.gov/drpseq/dwnld/Nikolaitchik_JV_2025_Fasta.zip.
